# Bridging Gaps in Adolescent and Adult Sexual Health: Training Medical Students as Educators

**DOI:** 10.7759/cureus.97769

**Published:** 2025-11-25

**Authors:** Abigail S Abraham, Sarah Nguyen, Avarie Willette, Zainab Farooq, Olivia Price, Paul Cervone, Elissa Barr

**Affiliations:** 1 College of Osteopathic Medicine, Lake Erie College of Osteopathic Medicine, Bradenton, USA; 2 Public Health, University of North Florida, Jacksonville, USA

**Keywords:** adolescent health, cultural competency, curriculum development, educator training program, medical education, patient-doctor communication, sexual health counseling, sexual health education, standardized curriculum, teaching and training residents and medical students

## Abstract

Background: The absence of a nationally standardized sexual health curriculum leads to inconsistent adolescent education on contraception, sexually transmitted infections (STIs), and other critical topics, leaving healthcare professionals the responsibility to fill this gap. Medical training also lacks standardized curricula in sexual health counseling; therefore many medical trainees report hesitancy in sexual health communication. This study evaluated the efficacy of a structured Sexual Health Educator Training Program in improving medical students’ confidence in sexual health communication, cultural competency, and legislative awareness.

Methods: In collaboration with the Florida Healthy Youth Alliance, 95 medical students at Lake Erie College of Osteopathic Medicine participated in a two-hour interactive training that included lectures, case-based scenarios, and collaborative discussions. Pre- and post-training surveys assessed confidence in eight domains of sexual health counseling.

Results: Confidence significantly improved across all measured sexual health counseling domains (p < 0.001). Students reported increased confidence in conducting sexual health conversations with adolescents and adults, counseling on contraception and STI prevention, and approaching discussions with cultural and religious sensitivity. On average, there was a 45% increase in students reporting high confidence across competencies.

Conclusion: Given the results of this research, Lake Erie College of Osteopathic Medicine - Bradenton has integrated the training into its Human Sexuality Course. This ensures the continued development of students’ competencies in sexual health communication and sets a precedent for other institutions to follow. These findings highlight the importance of standardized sexual health education training in equipping future physicians with the skills, cultural awareness, and confidence necessary for effective adolescent sexual and reproductive health counseling.

## Introduction

The absence of a nationally adopted sexual health education curriculum in the United States has led to inconsistent and inadequate adolescent instruction on contraception, sexually transmitted infections (STIs), and pregnancy prevention. As curricula vary widely across states and districts, significant knowledge gaps persist. The World Association for Sexual Health emphasizes integrating comprehensive sexual education into adolescent preventive care, yet many U.S. programs fail to address critical areas, including teenage pregnancy; lesbian, gay, bisexual, transgender, queer, plus (LGBTQ+) sexual health; and non-hormonal contraception options [1]. 

School-based sexual education remains politically and socially contentious. Requirements and content vary considerably across states, with many programs emphasizing abstinence while neglecting medically accurate or comprehensive instruction [2]. Abstinence-only approaches, designed to dissuade sexual activity, are directly correlated with higher rates of adolescent pregnancies and STIs [1,3]. Although most parents support sexual education, sociocultural and religious beliefs strongly influence what schools permit [4]. Consequently, some districts omit sexual health education entirely, leaving adolescents without reliable information.

These significant knowledge gaps leave the responsibility of providing adequate and comprehensive sexual health education to adolescents in the hands of healthcare professionals. However, medical professionals themselves receive little standardized training in sexual health communication, cultural sensitivity, or counseling on contraceptive options [5]. Effective counseling requires skills in communication and cultural awareness, yet these areas are frequently overlooked in medical education. Without such training, sexual health discussions can be uncomfortable for providers, leading to missed opportunities for guidance during adolescent visits.

To address this need, we implemented a structured Sexual Health Educator Training Program designed to strengthen medical students’ foundational competencies in sexual health communication. This study evaluates the program’s impact on students’ confidence across key counseling domains and explores its potential role within medical school curricula.

Objectives

The primary objective of this study was to evaluate changes in medical students’ confidence across eight domains of sexual health communication, counseling, cultural competency, and legislative awareness following participation in a structured Sexual Health Educator Training Program.

A secondary objective was to assess the feasibility and perceived usefulness of incorporating this training model into the existing medical school curriculum, with the broader goal of informing future approaches to standardized sexual health counseling curriculum within medical training.

Literature review 

Background

Sexual health education is essential for both individual and public health. Unfortunately, there is no standardized sexual health educator training for medical professionals, leaving providers underprepared and patients inadequately educated. This can have harmful consequences, particularly for vulnerable populations like adolescents, who may turn to online sources for information, risking exposure to misinformation [2]. This literature review examines the gaps that exist within sexual health education, particularly medical students’ exposure and training to handle conversations with patients about sexual health. This review focuses on the current state of sexual health education adolescents receive outside the clinic environment, the deficiencies in medical training, and the role of cultural competency in sexual health education. 

Adolescent Sexual Health Education Outside of the Clinic Environment

Sexual and reproductive health education in public and private schools remains a controversial and highly debated topic. There are no consistent teaching requirements regarding such topics, leading to many youth looking for answers to sexual health questions online or from their peers, ultimately subjecting them to misinformation. This section reviews the state of sexual education for youth to emphasize how essential it is for healthcare providers to be adequately trained to address and educate youth on sexual health during their visits. 

There is an inconsistency in sexual and reproductive health education provided to adolescents by schools in this country. Thirty-seven states require abstinence to be taught in sexual health education, while 25 mandate that abstinence be stressed and only 18 states require contraceptive information to be taught to adolescents. In addition, only 13 states require information taught during sexual health education to be medically accurate [2]. With only a small number of individuals mandated to receive proper options for contraception and medically accurate information, youth are vulnerable to misinformation both online and in the school education system. 

Research further supports the concern for adolescent sexual health education, as abstinence-only education has led to significant increases in teen pregnancy rates. Literature confirms that abstinence teaching does not correlate with absent sexual behavior, as it is positively correlated with teen pregnancy rates regardless of socioeconomic status, education level and ethnic background [1]. In addition, the US has the highest rates of sexually transmitted diseases among developed countries. This further supports the need for better sexual health education for youth to reduce teen pregnancy and sexually transmitted disease rates [1]. Since most schools do not provide adequate information, this responsibility falls on healthcare providers. 

Current Sexual Health Education in Medical Training

The current state of medical school curriculum surrounding sexual health education training varies greatly from institution to institution, as no current standardized training exists. A review of curriculum from 36 educational institutions found significant variation in the duration of training as well as training content [4]. Without standardization and metrics to assess, there is no assurance that physicians are able to fully address concerns of their patients, leading not only to significant differences in care but also to suboptimal care for patients. 

A study conducted by Beebe et al. of 276 medical students found that only 65.6% reported receiving formal sexual health education. Up to 20% of students received no training, and 13.9% received informal education during medical school. The lack of training negatively impacted the medical students’ confidence in discussing sexual health issues with patients [5]. This study emphasizes the need for standardized sexual health education training to increase confidence levels of future physicians so they can openly discuss, educate, and address any sexual problems that may be presented by patients. 

Without standardized health education training, even trainees who do receive sexual health educator training may be missing vital information that is needed to fully inform and educate their patients. Duane et al. examined over 20 medical school curricula and found that a large portion of the curriculum is devoted to topics like abortion procedures, birth control, and family planning, but lacks comprehensive coverage of alternative reproductive health approaches such as non-pharmacological family planning methods [6]. A broader approach to reproductive health education training is needed for medical students so they can offer and thoroughly inform patients of the broad range of reproductive health options available. 

In 1970, the University of Minnesota developed a comprehensive course in human sexuality in order to address the need for training within medical schools. Some topics covered in the course include communication with patients about sexual health, sexual health throughout the lifespan, and safe sex guidelines. A strength of this program is that it incorporates all aspects of sexual health across varying cultures and sexualities. However, the program does not have a mechanism to evaluate students’ understanding and incorporation of the material into their practice [7]. While the course created by the University of Minnesota is designed to build future physicians’ confidence in sexual health, it may not be widely implemented because there was no metric to examine the success of the course. Our study aims to address this gap by using a pre- and post-training survey given to students to evaluate confidence levels and the information gained from the training. 

"Medical and health care professionals’ sexuality education: state of the art and recommendations" highlights the importance of incorporating sexual health education into the healthcare curriculum [8]. They describe many professionals who report feeling their knowledge and comfortability are inadequate in addressing their patients’ sexual health concerns. In their review, Verrastro et al. examined the literature from 2000 to 2020 for studies pertaining to sexual health education for medical professionals. In one study examined, health care professionals attended a workshop that provided education and various resources they could share with patients all pertaining to sexual health. In a post-workshop survey, those who had attended the workshop were more likely to initiate discussions about sexual health with patients, 91% compared to 46% [8]. This review emphasized that training is necessary for the facilitation of an effective dialogue between providers and patients regarding sexual health. It highlights the success of programs that adequately train professionals and how this increased comfortability directly translates to the clinic environment. However, the study reports 79% of health care professionals felt they had inadequate training to address patients’ sexual health, leaving a significant gap that must be addressed. 

Deficiencies in Cultural Competency and LGBTQ+ Health

Cultural competency is vital to ensure patients of all backgrounds feel respected and valued by their healthcare provider. “Health workforce cultural competency interventions: a systematic scoping review” examines cultural competency training and interventions and how this affects practitioners’ knowledge and confidence providing care to those of diverse backgrounds. The review found that practitioners’ knowledge, skills, and attitude and beliefs were positively impacted by cultural competency training [9]. However, there is no standard measure of cultural competency training amongst medical students, leaving gaps in physicians’ preparedness for addressing these concerns. Cultural competency is especially vital when approaching the topic of sexual health, as various cultures and backgrounds have differing ideals and comfortability surrounding sex and reproductive health practices. A standardized training or assessment must exist to ensure sufficient and equal preparation to respectfully address sexual health issues for all patients. 

Cultural competency must include people of all diverse populations. Many previous studies have discussed a lack of training or confidence for medical professionals in educating LGBTQ+ individuals in their sexual health. In the article, “Bridging the gap in graduate medical education: a longitudinal pediatric lesbian, gay, bisexual, transgender, queer/questioning health curriculum” a LGBTQ+ health education program was incorporated into pediatric residency programs. The training included ​​didactic sessions, case-based discussions, and community engagement activities. When the effectiveness of the program was evaluated, they demonstrated improvement in provider comfort in asking about sexual orientation, gender identity, and sexual practices of patients measured through pre- and post-curriculum surveys [10]. The authors demonstrated success in incorporating LGBTQ+ training into graduate medical institutions and provided a framework for broader incorporation into all specialties and programs. 

Conclusion

Sexual health educator training is necessary for facilitation of an effective dialogue between providers and patients, yet no current standardized training exists for sexual health communication in the medical school curriculum. In addition, there is no standard measure of cultural competency training amongst health care professionals leaving a large gap that is yet to be filled. This lack of cultural competency is especially detrimental to LGBTQ+ individuals as they are often made to feel marginalized by healthcare. As there is no proper sexual health curriculum standard in schools, the knowledge gap regarding sexual health in adolescents becomes especially apparent when physicians also lack the skills to address reproductive health concerns. This study aims to bridge these gaps exemplified by a literature review in order to provide a standard training and assessment for medical students to be sufficient sexual health educators for their future patients. 

## Materials and methods

To evaluate the efficacy of the Sexual Health Educator Training Program, medical students at Lake Erie College of Osteopathic Medicine (LECOM) completed pre- and post-training surveys. The program was developed in collaboration with Dr. Elissa Barr, Professor of Public Health at the University of North Florida and founder of the Florida Healthy Youth Alliance (FHYA), a coalition promoting comprehensive sexual health education in Florida. The goal was to prepare students to serve as sexual health educators in clinical practice.

Training design 

The program consisted of a two-hour virtual session delivered via Zoom in lecture and discussion format. The session was facilitated by Dr. Barr, an expert in sexual health education and public health, who guided discussions, moderated case-based activities, and ensured consistency in the delivery of educational content. The training followed a structured sequence that integrated multiple instructional components to strengthen students’ communication, counseling, and cultural competency skills. The session began with an overview of adolescent sexual health trends, common clinical challenges, and gaps in school-based instruction. This was followed by a comprehensive review of contraceptive methods, including hormonal, non-hormonal, barrier, long-acting, and emergency options, with emphasis on how to counsel patients using individualized, patient-centered approaches. Participants also received an overview of Florida’s sexual health education legislation to contextualize the variability of school curricula and highlight areas where physicians may need to fill educational gaps.

Case-based scenarios allowed small groups to practice applying communication strategies, using inclusive language, and navigating culturally and religiously diverse patient situations. These scenarios provided opportunities to address sensitive discussions, model nonjudgmental counseling, and practice shared decision making. The session concluded with a large group debrief in which facilitators reinforced key concepts, answered questions, and demonstrated best practices in sexual health communication. Students were also provided with evidence-based resources and educational materials they could share with patients to promote sexual health literacy. 

Participants 

**Figure 1:**
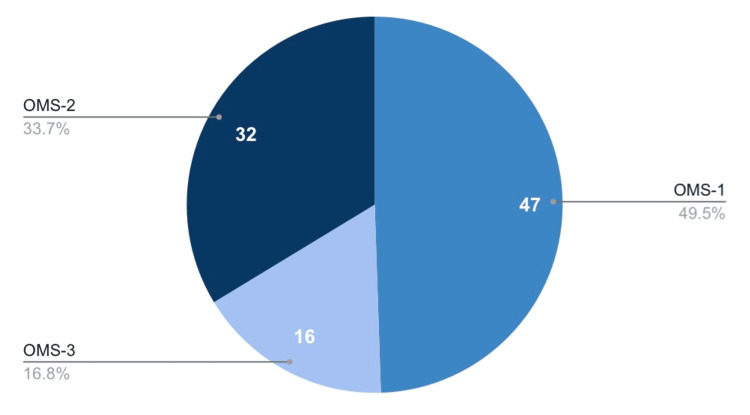
Distribution of Research Participants by Osteopathic Medical School (OMS) Year This figure displays the percentage distribution of participants across medical school years (OMS-1, OMS-2, OMS-3) who completed the Sexual Health Educator Training Program surveys. Nearly half of respondents were first year students.

**Figure 2:**
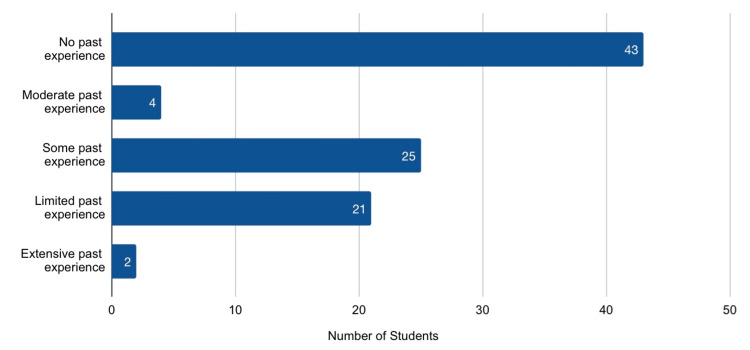
Prior Experience Discussing Sexual Health with Patients This figure illustrates the baseline experience of medical students in engaging in sexual health conversations with patients, ranging from no prior experience to extensive past experience. The majority of participants reported little or no experience prior to the training.

A total of 113 students completed the pre-training survey; 95 completed both pre- and post-training surveys, resulting in a matched dataset for analysis (completion rate: 84.1%). Nearly half were first-year students (47 osteopathic medical school (OMS)-1, 49.5%), followed by 32 second-year students (33.7% OMS-2), and 16 third-year (OMS-3, 16.8%) (Figure 1). Prior experience discussing sexual health with patients varied: 45.2% reported none, while only 2.1% reported extensive experience (Figure 2). 

Survey development

The survey instrument was developed by the research team in collaboration with faculty mentors experienced in sexual health education and medical education research. An initial item pool was created following an in-depth review of existing surveys measuring confidence and comfort in sexual health communication. The items were informed by domains used in previous research evaluating sexual health education interventions for medical students [11] and adapted to fit the specific objectives of this study. Draft questions were reviewed by research mentors for content validity, with feedback incorporated to ensure clarity, cultural sensitivity, and relevance to the training objectives. The final pre- and post-training surveys were developed by the authors to assess medical students’ confidence in sexual health communication, cultural competency, and legislative awareness. All survey questions were original and freely created by the research team; no copyrighted or licensed tools were used.

The survey included two types of five-point Likert-scale items [12]. Comfort-based items used a scale from 1 (“Not comfortable”) to 5 (“Very comfortable”) to measure students’ confidence in discussing birth control, STI prevention, adolescent sexual health topics, and sexual health concerns with patients from diverse backgrounds. Agreement-based items used a scale from 1 (“Strongly disagree”) to 5 (“Strongly agree”) to assess beliefs about the appropriateness of discussing sexual health during routine adolescent and adult visits, understanding of sexual health education techniques, and familiarity with Florida sexual health legislation. The survey also collected demographic information on year in medical school and prior experience discussing sexual health with patients. To support reproducibility, the full survey instrument is provided in the Appendix. 

Data collection

The surveys were administered electronically using Google Forms. The pre-training survey was distributed via email one week prior to the training session. Each student created a unique five-character ID to link pre- and post-surveys while maintaining anonymity. The purpose of the study and an informed consent statement were outlined in the introduction of the pre-training survey, and completion of the survey indicated consent to participate. The post-training survey was distributed immediately following the training session, and students were given 24 hours to complete it. The study protocol (Protocol 33-018) was reviewed by the Institutional Review Board of the Lake Erie College of Osteopathic Medicine and was determined to be exempt from further review. 

Data analysis

Paired t-tests were conducted to assess changes in mean confidence scores between pre- and post-training responses. Paired t-tests were conducted using Microsoft Excel (Office 365, Microsoft Corporation, Redmond, WA, USA). This analysis evaluated whether differences across all domains were statistically significant. A two-tailed p value of < 0.05 was considered statistically significant.

## Results

Confidence outcomes 

Paired t-tests revealed significant improvements across all eight domains (p < 0.001). Medical students’ confidence in discussing contraception and alternative options increased from 3.13 ± 0.75 to 4.46 ± 0.60 (t = -8.21, p <0.001), and in STI prevention from 3.29 ± 0.82 to 4.55 ± 0.65 (t = -7.92, p < 0.001). Confidence in discussing sexual health concerns with patients from various religious or sociocultural backgrounds rose from 3.04 ± 0.08 to 4.38 ± 0.62 (t = -8.10, p < 0.001). Confidence in guiding adolescents on puberty, sexual decision-making and safe sexual practices improved from 3.07 ± 0.78 to 4.48 ± 0.58 (t=-8.45, p < 0.001). 

Medical students also reinforced their belief that sexual health should be discussed routinely: in adolescence visits (increase from 4.12 ± 0.65 to 4.60 ± 0.55, t = -6.75, p < 0.001) and adult visits (increase from 4.28 ± 0.68 to 4.66 ± 0.57, t = -6.42, p < 0.001). Understanding of sexual health education techniques saw a substantial improvement from 2.92 ± 0.85 to 4.29 ± 0.70 (t = -8.30, p < 0.001), while the largest improvement was in students’ understanding of Florida’s sexual health education legislation (2.27 ± 0.90 to 3.78 ± 0.75 (t = -9.03, p < 0.001)). 

Overall change

**Figure 3:**
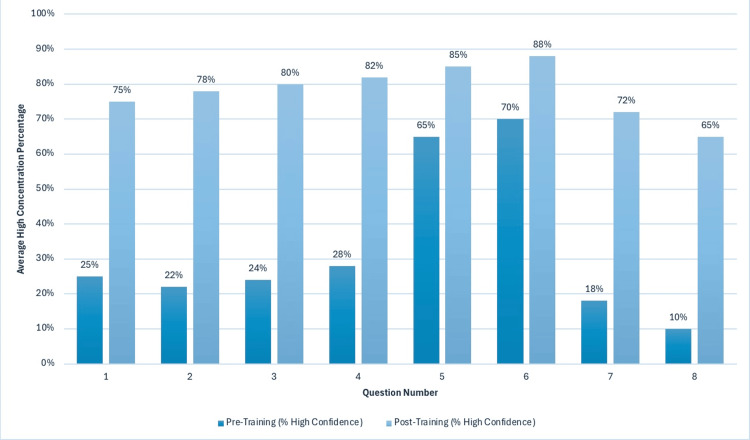
Response Distributions: Average Increase in Percentage of Students Reporting High Confidence on Likert Scale This figure presents the mean increase in high confidence responses across the eight survey domains. Data highlight the overall effectiveness of the training program in elevating medical students’ confidence in sexual health communication. Question numbers on x-axis correspond to question numbers in Table 1. Response Distributions: Increase in Percentage of Students Reporting High Confidence on Likert Scale [12].

**Figure 4:**
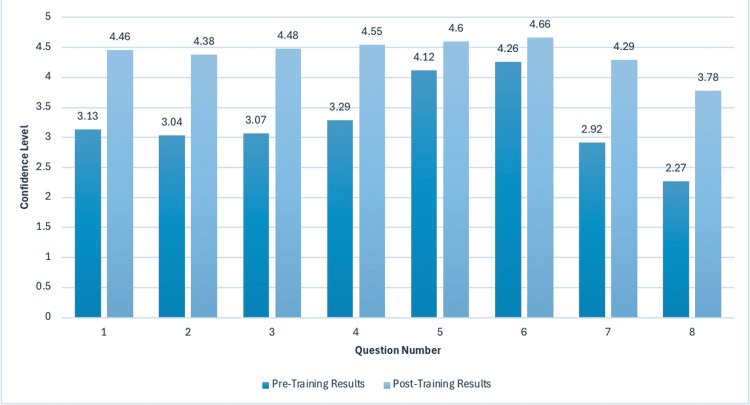
Response Distributions: Difference in Student’s Confidence Levels Per Question This figure compares pre- and post-training confidence scores for each survey question on a 5-point Likert scale. Results demonstrate statistically significant gains in all competencies, with the largest improvement observed in understanding Florida sexual health education legislation. Question numbers on x-axis correspond to question numbers in Table 1. Response Distributions: Increase in Percentage of Students Reporting High Confidence on Likert Scale [12].

Across all competencies, the proportion of students reporting “high confidence” (Likert scale [12] 4 or 5 ) rose by an average of 45.4%. Table 1 presents the percentage increase in students reporting high confidence within each domain. Figure 3 illustrates the average increase in high confidence levels per question, highlighting the greatest improvements in cultural competency and adolescent guidance on puberty and safe sexual practices. Figure 4 displays the overall shift in average confidence distributions across all questions.

**Table 1:** Response Distributions: Increase in Percentage of Students Reporting High Confidence on Likert Scale This table shows the percentage increase in students rating themselves as highly confident (4 or 5 on a 5-point Likert scale [12]) in various sexual health competencies from pre- to post-training. Substantial improvements were observed across all domains, with the greatest increases in adolescent counseling and culturally sensitive discussions. All survey items were author developed and freely available for academic use.

Survey Question	Pre-Training (% High Confidence)	Post-Training (% High Confidence)	Improvement
1. Comfort Discussing Birth Control Options	25%	75%	50%
2. Comfort Discussing Sexual Health with Diverse Patients	22%	78%	56%
3. Confidence Guiding Adolescents on Puberty & Safe Practices	24%	80%	56%
4. Comfort Discussing STI Prevention	28%	82%	54%
5. Belief That Sexual Health Should Be Discussed in Adolescent Visits	65%	85%	20%
6. Belief That Sexual Health Should Be Discussed in Adult Visits	70%	88%	18%
7. Understanding of Health Education Techniques	18%	72%	54%
8. Understanding of Florida’s Sex Education Laws	10%	65%	55%

## Discussion

This study demonstrates that a structured Sexual Health Educator Training Program significantly improved medical students’ confidence across all measured competencies. Pre- and post-survey results revealed consistent gains in communication skills, cultural competency, and legislative awareness. Improvements clustered around three critical domains: attitudes, knowledge, and skills [13]. Together, these findings underscore the value of standardized training in strengthening medical education and preparing students for effective sexual health counseling.

Attitudes 

Confidence in discussing sexual health with patients from diverse religious or cultural backgrounds increased by 56%, reflecting the program’s emphasis on inclusivity. Prior research links increased cultural awareness to stronger physician-patient relationships and improved adherence [9]. Our case-based approach provided exposure to sociocultural considerations while encouraging reflection on personal beliefs and ethical perspectives, aligning with broader models of cultural competency training that promote spaces for self-reflection on personal beliefs and perspectives, ethical considerations, and the understanding of variations in human sexual expression and identification [13]. Although cultural competency is a skill that develops over time, early exposure through structured training fosters confidence and lays the foundation for respectful, patient-centered communication. 

Knowledge

This training resulted in a 56% increase in participants’ comfort and understanding of guiding adolescents on puberty, sexual decision-making, and safe practices, which translates into clinical practice by encouraging more frequent and open conversations - an approach shown to reduce adverse health outcomes [14]. This is a critical gain given persistent U.S. rates of unintended pregnancy and STIs. Comprehensive adolescent education is associated with healthier behaviors such as condom use and reduced risk-taking [14]. Previous studies have shown that although confidential adolescent encounters remain an opportunity to educate, communication between providers and their patients is still limited and must be strengthened [15]. Therefore, by strengthening medical trainees’ knowledge in these discussions, the program may promote earlier, more effective preventive counseling during clinical encounters.

Skills

Confidence in contraceptive counseling increased by 50%, while STI prevention counseling improved by 54%. The literature demonstrates that the largest gaps in medical students’ knowledge exist in topics such as “safety and prevention, sexual minority health, and fertility and reproduction” [16]. However, studies demonstrate that workshops and structured training increase provider initiation of sexual health discussions [8]. Therefore, by emphasizing both counseling skills and education techniques, this program addresses one of the most persistent gaps in medical education. 

Legislative awareness

The most dramatic improvement was seen in participants’ understanding of Florida’s legislation on sexual health education in the school systems. High-confidence responses rose from 10% pre-training to 65% post-training - a 55% gain. It was imperative for the medical students to understand the current state of Florida’s sexual health education curriculum as it underscores the educational gaps within the state’s school systems. Awareness of policy gaps allows providers to anticipate what patients have (or have not) learned in schools and tailor their counseling accordingly. In politically polarized environments, this skill is vital for physicians tasked with bridging inconsistent education. 

Comparison with other programs

While training modalities vary widely, our program’s fully virtual format increased accessibility for both pre-clinical and clinical students. During this program, three main goals were addressed: knowledge, attitudes, and skills. Knowledge gaps were addressed by reviewing behaviors, health outcomes, and Florida legislation. Attitudes were explored through discussions on approaching conversations with cultural and religious sensitivity, while skills were strengthened through case-based exercises requiring application of learned strategies.

This approach reflects just one of many educational models described in the literature. Other interventions include single-session lectures with practice components, workshops combining didactic and role-play, chart review exercises, and longer seminars spanning several weeks or an entire semester [4]. Formats also range from synchronous to asynchronous or blended learning environments. Furthermore, evaluation metrics differ considerably across programs, ranging from knowledge, comfort, and attitudes to the self-efficacy of the training itself [4].

Our program contributes to this field by combining accessibility, comprehensive domain coverage, and structured evaluation, offering a scalable model that could be adapted nationally.

Broader implementation and adaptation

While structured sexual health training offers clear benefits for medical students, national standardization would require flexibility given the substantial variation in state policies and political climates. A competency-based framework, rather than a mandated uniform curriculum, may provide a more feasible approach, allowing institutions to adapt core learning objectives such as communication techniques, cultural humility, and contraceptive counseling within their local contexts.

This training model could also be adapted for other health professional groups, including residents, physician assistant students, public health students, and nursing students, by tailoring case complexity, emphasizing discipline-specific communication challenges, and integrating interprofessional collaboration exercises. Such modifications would allow the curriculum to remain scalable while meeting the unique needs of diverse healthcare learners.

Limitations 

While this study demonstrates significant results, a few limitations remain. The survey relied on self-reported Likert-scale ratings [12], which measure perceived confidence rather than objective counseling skills, and the instrument was not psychometrically validated. The single-arm pre-/post-training design without a control group limits causal interpretation, and the post-training survey captured only short-term outcomes. Additionally, the study was conducted at a single institution with limited demographic data, affecting generalizability. Future research should incorporate objective measures, multi-site samples, and longitudinal follow-up.

## Conclusions

The lack of standardized sexual health education for adolescents, combined with inconsistent training for medical professionals, has created substantial knowledge gaps in sexual health overall that must be addressed. These gaps contribute to risky behaviors, misinformation, and neglect of the unique needs of diverse adolescent populations. As school-based education remains limited or contested, physicians are increasingly responsible for filling this void. However, there is considerable variation in how sexual health education and communication are addressed in medical training curricula across institutions. 

Our findings demonstrate that medical students enter training with low confidence in sexual health counseling but show significant improvement following a structured Sexual Health Educator Training Program. Post-training, students reported marked gains across all domains: attitudes, knowledge, skills, and legislative awareness, highlighting the program’s effectiveness in building confidence for future clinical practice. While these results indicate that a structured training program can increase medical students’ perceived preparedness to engage in sexual health discussions, further research using objective assessments and longitudinal follow-up is needed to determine the extent to which these confidence gains translate into clinical competence

Given the success of the training, Lake Erie College of Osteopathic Medicine has incorporated the Sexual Health Educator Training into its medical school curriculum, ensuring the continued development of students’ competencies in sexual health communication. By setting a precedent for curricular integration, this study offers a model for other medical institutions nationwide to adopt a structured sexual health training, ensuring that future physicians across the nation are confident in their critical role in sexual and reproductive health counseling.

## Appendices

Sexual health educators pre- and post-training survey instruments

All pre- and post-training survey items were administered electronically via Google Forms. The post-training survey contained the same items as the pre-training survey to allow direct comparison of confidence and agreement ratings.

Participant Information:

1. Personal ID

Pre-training: “Create your ID by listing 1 letter and 4 numbers (e.g., A1311).”

Post-training: “Please use the same personal ID from your pre-training survey.” (Short answer response)

2. What OMS year are you in?

3. I have past experience engaging in discussions on sexual health education with patients.

No past experience

Limited past experience

Some past experience

Moderate past experience

Extensive past experience

Comfort and Confidence Items (Likert Scale 1-5):

(1 = Not comfortable, 2 = Slightly comfortable, 3 = Moderately comfortable, 4 = Comfortable, 5 = Very comfortable)

4. I feel comfortable discussing birth control options and alternatives with patients.

5. I feel comfortable discussing sexual health concerns with patients from various religious or sociocultural backgrounds.

6. I feel confident providing guidance to an adolescent on topics such as body changes during puberty, sexual decision-making, and safe sexual practices.

7. I feel comfortable discussing protective and preventive factors regarding sexually transmitted infections with patients.

Agreement-Based Items (5-Point Agreement Scale):

(1 = Strongly disagree, 2 = Disagree, 3 = Neutral, 4 = Agree, 5 = Strongly agree)

8. I believe sexual health concerns should be a topic of discussion during routine adolescent/young adult patient visits (patients less than 18 years old).

9. I believe sexual health concerns should be a topic of discussion during routine adult patient visits (patients ≥18 years old).

10. I understand how to utilize health education techniques to create an open dialogue with patients regarding safe sexual practices.

11. I understand what Florida legislation states regarding sex education.
